# Creative Mental Health Literacy Practices: A Qualitative Study Exploring How Students Use Literacy to Promote Wellbeing and Manage Mental Health Conditions While at University

**DOI:** 10.3390/ijerph20156475

**Published:** 2023-07-31

**Authors:** Emily Peach

**Affiliations:** Department of Linguistics and English Language, Faculty of Arts and Social Sciences, Bailrigg Campus, Lancaster University, Lancaster LA1 4YW, UK; e.peach1@lancaster.ac.uk

**Keywords:** mental health, literacy, university students, creative practices, reading, creative writing, bullet journaling

## Abstract

Understanding how students manage their mental health while at university is more important than ever, given the increasing number of undergraduate students experiencing poor mental health and seeking support from their universities. This paper reports on an exploratory qualitative study and discusses how students with mental-health conditions use literacy (reading, writing, and the use of texts) to manage their mental health, focusing on reading for pleasure, creative writing, and bullet journaling. Through in-depth qualitative interviews across the academic year of 2018–2019, 11 students’ reflective accounts of their practices in managing their mental health were collected and then thematically analysed. This paper focusses on the experiences of three students as case studies of how students use literacy to manage times of mental health distress and promote wellbeing through relaxation, the expression of emotions, and the recording of their mental health. These practices enabled the processing of emotions, engagement in supportive relationships, development of a sense of self, and reflection of mental health progress. These findings demonstrate that supporting students to engage in self-directed creative literacy practices could help students to manage their mental health and develop on-going positive strategies while helping universities manage the increased demand for help from students.

## 1. Introduction

The relationship between literacy (reading, writing, and the use of texts) and mental health has been a topic of interest for many years for researchers, public mental health professionals, and people with mental-health conditions [1,2]. This paper seeks to add to the existing understanding of how people can use literacy practices to manage their mental health and promote wellbeing through addressing current gaps. Much work has focused on facilitated interventions in therapy settings [3,4,5], rather than the self-directed practices of individuals. The research question answered in this paper, which uses data from a wider study, is: how do students with mental-health conditions use creative literacy practices to manage their mental health? Thus, this paper focused on what young people with mental-health conditions do with their own self-directed creative literacy practices to manage their mental health from their own perspectives. Furthermore, there has been limited previous research exploring how students at universities look after their mental health through engaging with creative practices, including literacy practices. 

Firstly, in this introduction, the research context is further explained. The current situation in student mental health is summarized, highlighting the increasing levels of poor mental health in this population and the growing demand for support. Relevant theory on the approach to mental health and literacy is then briefly outlined. Secondly, the research approach, questions, and methods are presented, including information on the participants, data collection, and data analysis. Then, the experiences of three students are described as case studies of the different ways that students use literacy practices to manage their mental health while at university, before being discussed in terms of previous research. These experiences show how creative literacy practices can be beneficial for students’ mental health, providing opportunities for relaxation, allowing for processing and releasing emotions, and enabling the reflection and recording of mental health progress. Further directions for future research are outlined before conclusions are drawn in Section 5. 

### 1.1. Student Mental Health

In the United Kingdom, student mental health has been a topic of much discussion in recent years, drawing attention from official bodies [6] and the media [7,8]. These reports have focused on the increasing numbers of students experiencing poor mental health and distress during university study and the level of support being provided by universities. It is difficult to accurately ascertain levels of poor mental health in the UK student population as there are limited longitudinal data [9,10]. Higher Education Statistics Authority (HESA) annual data [11] showed an over eight times increase in the number of first year students registering a mental-health condition as a disability upon entry to university (0.5% in 2009/2010 and 4.32% in 2019/2020). However, this is unlikely to represent the full extent of the situation. The increase in registering mental health as a disability may also reflect increased awareness.

Academic studies of student mental health suggest there are high levels of poor mental health in student populations [12,13,14,15,16]. For example, McLafferty et al. [12] reported a 47.5% 12-month prevalence rate for mental-health conditions in their 700 first year student participants and Farrell et al. [13] reported that 89% of their Welsh medical student participants had experienced mental health distress. Research undertaken by Chen and Lucock [17] with over 1000 students during the COVID-19 pandemic found that 53.4% had clinical levels of depression and 51.5% had clinical levels of anxiety. The reasons for the current levels of poor student mental health are multiple and complex. In the UK, financial pressures on students have been increasing in recent years with changes to tuition fees and the student loan systems [18], as well as an ever-growing focus on employability [9,14,19]. Studying at university is also a period of transition for many students [20], for example with moves away from home and support networks, which can lead to homesickness [21,22,23], and moves into higher-level studies with associated assessments and the cycle of submission and feedback [20,24,25,26]. 

University can further be a time of identity exploration, including a development into emerging adulthood, which can lead to stress and mental health pressures [27,28,29,30]. In Australia, survey research suggested that higher education students experienced higher levels of moderate mental health distress compared with non-student peers, but that there was little difference in severe mental health distress [31]. Further research based on similar survey data in Australia found that when viewed longitudinally, the situation may be more complex [32]. Over time, students seemed to have better mental health in general when compared with their non-student peers [32]. Research in the UK also found evidence that students may have lower levels of psychological distress than their non-student peers, with authors suggesting this may be due to the level of mental health support accessible from universities [33]. 

However, there were key moments where students experienced worsening of their mental health when compared with their non-student peers, which were identified as important transition periods for students, including final exams before and during university [32]. Taken together, previous research on student mental health suggests both on-going and acute pressures for students. This means that students need to be supported in developing on-going strategies to manage their mental health, which can also be drawn on in times of acute stress, such as assessment and transition into further study [25]. 

Support services at universities in the UK have been heavily impacted by the increased demand for support arising from high levels of poor student mental health [34]. In the 2010s, significant increases in the number of students seeking help were reported [34,35]. Recent work on student help seeking has reported continued high levels of help-seeking by students in the UK; for example, Cage et al. [25] and Batchelor et al. [36] found that 55.9% of their student participants had sought support for their mental health while at university. Increasing demand for support is complicated by more students seeking support for more complex problems [37]. This increasing demand for support has not been matched by an increase in resources or funding for mental health services at universities [16,38]. Instead, there has been a shift in therapeutic approach to very short-term counselling [38,39] and a move to entire university approaches to mental health [40]. In this context, research exploring the ways that students support their mental health while at university is even more necessary, to ensure that universities support all students in developing effective management strategies. Research co-created with students has demonstrated that they want more information on preventative and positive wellbeing strategies and want this to be a priority for research into student mental health [28,41]. 

### 1.2. Mental Health and Literacy as Social Practice

Mental health is a complex concept. Thorley [10] proposed thinking of student mental health in terms of two related continua. The first continuum, mental health, is understood to be something that all people always have and that can be more positive or more negative dependent on an individual’s symptoms, level of distress, thoughts, and related behaviours [10]. The second continuum, wellbeing, is understood as “the extent to which an individual is feeling good and functioning positively” (p. 9, [10]). While there is a relationship between mental health and wellbeing, Thorley [10] argued that people with negative mental health (i.e., a mental-health condition or illness) can have high levels of wellbeing for periods of time and people with positive mental health (no on-going symptoms or conditions) can have periods of low levels of wellbeing. This understanding helps to capture the fluctuating and on-going nature of students’ mental health issues while at university [10]. 

Literacy is understood in this research from the perspective of Literacy Studies. Literacy Studies is a multi-disciplinary field that views reading, writing, and the use of texts as social practices that are purposeful, contextual, and shaped by power relations [42]. This perspective has been fruitful in exploring people’s lived experiences and the role of literacy in their lives, including with health [43,44]. The social practice approach to health literacy focusses on the socially and contextually embedded, purposeful literacy practices used in the domain of health [43,44,45]. In my work, I propose taking a social practice approach to mental health literacy as the situated literacy practices used by individuals in the domain of mental health and to engage with their mental health on any level [46]. The social practice approach I propose allows for the collection of detailed, nuanced, and contextualized accounts of people’s actual lived experiences in using literacy to navigate mental health support systems and manage their mental health.

### 1.3. Literacy for Mental Health

The potential for reading and writing to play a role in mental health treatment and management has been explored in previous research. This overview is going to focus on literature concerning reading, creative writing, and bullet journaling. 

#### 1.3.1. Reading for Mental Health

Research on reading for mental health appears to have been largely focused on bibliotherapy, with some research on reading for pleasure. Bibliotherapy is a therapeutic approach where a therapist or facilitator prescribes a particular book or poem to an individual or group with the aim of fostering emotional processing, personal development and growth, or reflection on an experience [4,47]. There is a long history of using literature for self-development and reflection, but modern understandings of bibliotherapy emphasize the need for facilitation by a trained professional and the combination of reading with particular reflective activities, usually in a group [3,4,48]. Although this focus on formal facilitation and reflection differs from the self-directed reading practices discussed in this paper, research on bibliotherapy still has relevance, particularly in the description of how bibliotherapy participants experience reading for their mental health. 

Beyond research on facilitated bibliotherapy as an intervention, there has been some research exploring reading for pleasure, emotions, and wellbeing. Much of this research has been conducted by librarians or educators and has focused on educational settings [49,50,51,52]. There has been a recent study exploring reading for pleasure (described as recreational reading) and wellbeing in university students [53]. Levine et al. [53] used surveys to capture the recreational reading habits and psychological distress levels of university students at different points throughout an academic year. They found that higher levels of recreational reading were associated with reduced levels of psychological distress, including decreased symptoms of anxiety and depression [53]. The reduction in psychological distress was attributed by the authors to the way that reading allowed students to feel connected to a fictional world and enabled improvements in communication skills [53]. Levine et al. also emphasized that the volitional nature of recreational reading seemed important in terms of the positive effects on psychological distress [53]. 

Across the literature on bibliotherapy and reading for pleasure, there has been further discussion on the mechanisms underlying the positive effect of reading on mental health or wellbeing. Relaxation has been identified as an important aspect of reading for pleasure [51,54,55,56], with this relaxation being one of the primary positive effects on mental health for readers [3,57]. Dowrick et al., for example, described how the reading group participants in their study would visibly calm when reading, becoming less restless and physically stiller, with participants reporting reduced mental anxiety and being soothed by reading [57]. It has also been suggested that reading is immersive, which provides an escape from everyday life [3,52,54,56,58]. In Nell’s classic study, for example, participants described their reading as being an “island of delight” (p. 43) away from a mundane and difficult life [58]. Reading can also be an opportunity for making or finding meaning, both in relation to the inherent meaning of the text and how the text links to personal experiences or identities, which can be beneficial through encouraging deep focus, reflection, and self-development [48,52,57,59]. 

#### 1.3.2. Writing for Mental Health

Turning to writing and mental health, there have been a range of different therapeutic approaches developed involving writing, including expressive writing [60], fictionalized autobiography [61], control writing [62], and Using Writing as Therapy [63]. Expressive writing, which has been the focus of much research on writing as therapy [1], centres on the expression of inner emotions, personal truths, and secret traumas, with writers expected to write as themselves, for themselves, and about true and real events [60,64,65]. Creative writing, on the other hand, involves writing fictionally, including voice, characterization, plot, narrative, an imagined audience beyond the writer, and aesthetics [1]. There has been minimal discussion in the literature of creative writing as a therapeutic approach [1,2,66]. Two key studies have focused on group therapeutic interventions using creative writing [1,67]. King et al. [67] explored the potential therapeutic benefits of creative writing for people with serious mental illnesses (e.g., schizophrenia), finding that creative writing could help individuals develop a more coherent narrative about their identities and lives, as well as help address cognitive declines associated with serious mental illness. Deveney and Lawson [1] reported on a study investigating the effects on wellbeing of a series of group creative writing classes. They found that creative writing helped their participants express and process emotions, find catharsis, explore trauma and find positives from difficult experiences [1]. 

As Costa and Abreu identified, the terms used in the literature when discussing therapy and writing can be inconsistent and the boundaries between different approaches can be unclear [2]. In the current literature, poetry therapy seems to be treated as a distinct approach to creative writing as therapy. Poetry therapy focuses on the reading and writing of poetry for emotional processing and catharsis [68,69]. As with creative writing, much of the research on poetry therapy discusses group interventions [70,71,72,73,74] or personal narratives of individuals’ use of poetry for managing emotions or promoting mental health [68,75]. Anderson et al. [71] identified some of the characteristics of poetry that can be beneficial for mental health, namely aesthetic value, rhythm, universality of emotions, catharsis, and literary techniques. 

There is limited research on bullet journaling, including in relation to any potential relationship with mental health, but there has been some discussion in the field of informatics and human-cantered computing. Bullet journaling involves creating layouts in physical notebooks, which are often artistic and visually appealing, to record and display different types of information [76]. Tholander and Normark [76] conducted social media observations and qualitative interviews with people who kept bullet journals. They found that bullet journaling allowed participants to assess different situations in their life, reach new perspectives, engage in self-creation and reflection on their lives, accept imperfections more readily, and resist discourses of productivity [76]. The potential benefits of the practice on users’ mental health were not fully explored by Tholander and Normark, but they did report that bullet journal users could engage with the practice in order to improve their health or wellbeing [76]. Ayobi et al. [77] also studied social media data relating to bullet journals, finding that users were creative in how they designed and combined information. The analysed posts showed that users reflected on their bullet journal designs and this allowed for wider reflection, as well as on their life, and that users engaged in practices of self-tracking through their bullet journal, including mood and other mental and physical symptoms [77]. The authors suggested that the flexibility and mindfulness of bullet journaling provided users with an effective way of meeting their emotional needs and engaging in positive and therapeutic thinking [77]. 

## 2. Materials and Methods

The data discussed in this paper come from a wider research project exploring the literacy practices of students with mental-health conditions. The research was approved by the Lancaster University FASS-LUMS Ethics Committee. The ethical processes followed in the research have been discussed in detail elsewhere [78]. Participants were self-selecting and informed consent was sought. They were given opportunities to withdraw throughout the research process, as well as after data collection had finished. The use of video-calling interviews allowed the participants and researcher to be in physical spaces where they were comfortable during the data collection, which helped balance the risks to participant and researcher safety, with the need for discretion and privacy when discussing potentially sensitive topics [78]. Including video alongside audio enabled the researcher to assess participants’ emotional state through observing body language and facial expressions [78]. Participants were reassured at the start of each interview that they could end the interview at any time and that they could opt out of any questions or topics with which they were uncomfortable. At the start of the research process, sources of support for each student were identified in case they felt distressed during or after an interview. All of the participants were provided with information on sources of further support and had a debriefing with the researcher at the end of the data collection period. 

The wider project on the literacy practices of students with mental-health conditions investigated the following three research questions:

1. How do students with mental-health conditions use literacy practices to navigate support systems, both inside and outside the university?
2. How do students with mental-health conditions use literacy practices to manage their mental health, including in relation to their academic work?
3. How do students with mental-health conditions experience being a student, particularly their academic work and writing for assessment practices?

This paper focusses on the data collected in relation to the second research question on the management of mental health using literacy, where some of the students shared that they used creative literacy practices as part of managing their mental health using literacy. The initial research questions for the project were developed based on previous work on health literacy from a social practice perspective [43]. 

A qualitative approach to research adopting an ethnographic stance was taken [79] in order to allow for the capture of contextualized data representing the participants’ real experiences with literacy and their mental health. Gillen and Ho (p. 40) explained that an ethnographic stance requires a “commitment to uncovering the emic, deploying a number of research methods, as appropriate, and considering the role of the researcher” [79]. In this context, an ethnographic stance involved cantering participant experiences, for example, by using participant contributions to shape later data collection and being reflexive of my own positioning as a researcher, teacher, and past student with a mental-health condition. As a result of issues of access and confidentiality, there was no participant observation as would be necessary for a fully ethnographic study, following previous research on health literacy from a social practice perspective [43]. Given the sensitivity of the research and the limited access to observation opportunities, data collection involved collecting participants’ reflective accounts through in-depth interviews [43]. Focusing on collecting participants’ reflective accounts allowed for mental health experiences, support, and treatment to be discussed in ways that prioritised participant comfort and enabled participant control of my access to their information and experiences. Data collection occurred across the academic year of 2018–2019, comprising of a series of three interviews: an initial guided interview followed by two semi-structured interviews based on earlier participant contributions [80]. The initial guided interview focused on obtaining background information about the participants with topics chosen based on previous research on health literacy [43]. Table 1 shows the interview plans for each of the three stages. Across all stages, these plans were used as guides rather than strict schedules. In all interviews, the participants’ contributions drove the conversation, allowing for their own perspectives to be centred [80,81]. The interviews were undertaken using video-calling software to help resolve the tensions in this ethically complex research (for further details of the ethical complexity in this research see [78]). I used Skype to conduct the interviews, and audio recorded the interviews using a low-cost third-party software [82]. These recordings were then encrypted before being transcribed and the data were anonymized. 

Eleven students were recruited for the project, all of whom identified as having a mental-health condition. The participants were self-selecting and were recruited through advertising on campus and on relevant student online noticeboards. The majority were undergraduate students (9) from the UK (7); further details about the participants are provided in Table 2. As the wider study was exploratory, not all of the participants discussed using creative literacy practices for mental health in detail. Some of the participants (5) did not discuss using creative literacy practices in any way to manage their mental health. Six participants did discuss using creative literacy practices in some way as part of their mental-health management. In this paper, three participants, Amelia, Josie, and Gabriella, were the focus, as their reflections on their use of creative literacy practices best exemplified the themes identified during the analysis. The other three participants who discussed creative literacy practices to some extent were Jordan (comedy writing and script writing), Rebecca (writing a blog and playing video games), and Eva (reading novels about mental health). The decision was made to focus on only three participants in this paper in order to allow for greater depth, nuance, and complexity in the analysis. Analysis of the full data set can be found in author’s thesis [46].

Josie, Amelia, and Gabriella (pseudonyms) were all female undergraduate students from the UK, aged between 18 and 20. Josie was a second year student in the social sciences who identified as having anxiety and depression. She had no history of seeking help for her mental health before university, but had experienced some difficulties with her mental health throughout her teen years, although this was something she only realized when she learnt more about mental health once arriving at university. Amelia was a first year student in the humanities who identified as having depression and anxiety, and also described herself as having autism spectrum disorder. She had a long history of poor mental health before university and was experienced in accessing support and using techniques to manage her mental health. Gabriella was also a first year student, studying for a degree in the arts and identifying as having anxiety and depression. Since Gabriella had been at university, her family and friends had shared concerns surrounding self-harm and suicidal ideation with the university. This had led to referrals into the university’s counselling and mental health services. She had sought little help for her mental health before arriving at university, but now was trying to engage with support providers. 

### Data Analysis

A thematic analysis approach was taken for the data [83], with coding undertaken using NVivo 11 Plus [84]. Initial codes were generated based on familiarization with the data, before more coherent themes were developed. These themes were taken to the whole data set, reviewing the patterns and coherence of the data and the naming of the themes [83]. This review resulted in a reworking of the themes, with the final themes being assessed again across the whole data set and being used to choose extracts of data for writing up. Throughout this process, the raw data were continually re-visited to ensure that the focus remained on the students’ experiences and voices. The data analysis was discussed during supervision sessions to help ensure that the analysis was reflective of the participants’ contributions. This involved reviewing analysed transcripts together, discussing the development and revision of codes and themes, and debating which data extracts best fit the themes for writing up. 

## 3. Results

### 3.1. Reading for Mental Health: Josie’s Experience

When talking to Josie about how she managed the symptoms of her mental-health condition, she explained that reading for pleasure was one of the primary tools she had for coping. Reading was a long-standing hobby for Josie, which she continued to engage with after arriving at university. For Josie, reading was part of a “whole process that relaxes her a lot”, involving “the smell of (a physical book) and the feel of holding a book”, “get(ting) a cup of tea (…) and (her) blanket”. These embodied elements worked together with the act of reading to help Josie relax and “wind down”, which helped her manage during periods of high stress or anxiety. Josie’s choice of reading materials was usually fantasy or young adult novels because they contrasted the more serious and dense texts she read for her studies. These genres also provided a greater level of escapism for Josie as she became “invested” in the plot and could immerse herself in very different worlds. Josie used this escapism to distract herself from distressing symptoms and provide herself a break from the reality of her current situation. 

Reading for pleasure also provided Josie with other positive benefits for her mental health. Josie explained that reading for pleasure to relax in this way, with the wider ritual of the physical book, tea, and blankets, was something she had done “since (she) was tiny” as she “had got it off her mum”. Josie’s continued use of this practice seemed to be a way of drawing on the supportive relationship she has with her mother, which was now long-distance after Josie’s move to university. This entwining of Josie’s reading practices with her relationship with her mother could further be seen in Josie’s description of both herself and her mother as being “bookworms”. Being a “bookworm” was an identity Josie seemed proud to claim. Reading for pleasure, for Josie, in addition, seemed to be a way of engaging with her sense of self and promoting her self-esteem. She was proud of being a “bookworm” and felt good about herself when she cemented this identity by reading. Reading for pleasure publicly enabled Josie to also express this identity to others and project a particular image of herself, one which she liked and was proud of:

“It’s like if I get my phone out on like a train, people will be like oh what is she doing or like, oh god another teenager just on social media that kind of thing but like when I’ve got my book I feel like (a) I’m more invested in it than social media and (b) it also doesn’t look so anti-social I guess, I don’t know I just prefer that side of things, the image it gives off yeah”.

The relationship between literacy and identity is embedded in social relations; the way an individual is perceived and wants to be perceived by others plays a role in how they form a sense of self [85,86,87]. Through reading for pleasure in public and thus presenting herself as a “reader” or “bookworm” to others, Josie may have also improved her own image of herself, thus boosting her self-esteem. 

### 3.2. Creative Writing for Mental Health: Amelia’s Experience

Amelia was very experienced in managing her mental health by the time she arrived at university. She was interested in mental health as a topic and read self-help books to try and learn more about why she felt the way she did and understand her symptoms and conditions better. One of the strategies Amelia had learned was helpful was creative writing, and specifically writing poetry. Amelia often wrote what she termed “vent poetry” when she was having distressing thoughts or feeling distressing emotions. This “vent poetry” was like spoken-word poetry in terms of style and format and the content was focused on her experiences. 

When asked how writing the poetry helped her manage her mental health, Amelia explained that writing about her emotions and experiences provided her a sense of relief. She described how writing her poetry down felt like she was getting her thoughts and feelings out of her head and onto the page, giving a sense of physical relief. Through the process of writing, Amelia was able to achieve some catharsis, which was positive for her mental health.

Beyond the catharsis Amelia felt when writing, she also explained how writing the poetry allowed her to process her emotions to some level of resolution and gain a more objective perspective on situations and experiences. She explained that the processing came through the flow of her writing and the need to consider which literary techniques were most suitable to represent her thoughts or feelings. Thinking about how best to articulate what she was thinking or feeling in an aesthetically pleasing way to an imagined audience required Amelia to detach herself from the immediate thoughts and feelings and consider them from a more distanced perspective. This helped Amelia feel more in control of her emotions, which was empowering and positive for her mental health. Amelia further explained that the aesthetic element of creative writing had benefits for her mental health. She described finding it helpful to see her distressing emotions and thoughts turned into something “beautiful” through her poetry writing. This lessened the emotional distress Amelia felt and helped her manage her difficult emotions. 

Amelia used her poetry to manage her mental health beyond the initial writing process. She saw her poems as resources that she could revisit later; for example, if she experienced the same emotion or thought again. When she revisited a poem, she would often refine her writing to better communicate her thoughts and feelings or to be more aesthetically pleasing. This helped her further develop a more objective or critical perspective on her experiences, which she found helped her develop a better understanding of her emotions and mental health symptoms. Amelia also used her poems as a resource that she could read, rather than work on again. The poems served as reminders of the creative writing process and allowed her to relive the positive effects the process had previously had on her mental health. She was also able to compare her current situation to the situation she had captured in a poem, this helped her identify the progress she had made and reflect on changes in her life and mental health. 

### 3.3. Bullet Journaling for Mental Health: Gabriella’s Experience 

Gabriella was in the process of learning about her mental health and developing new strategies for managing her mental health more effectively. She had been bullet journaling for some time before university, but had started to use the practice more intentionally for her mental-health management. Gabriella used her bullet journal to record a range of different things, including planning out her schedule, making notes for her academic work, tracking her water intake and keeping a daily tracker for her moods. The layout of this daily mood tracker was designed by Gabriella. Across a double page spread, Gabriella drew a calendar for the week. Then, each day Gabriella would colour in a space on the calendar with a different colour depending on her mood for the day. She explained that she found the process of both drawing the layout at the start of the week and then colouring in a box each day to be relaxing and satisfying. Expressing her moods visually was helpful as it allowed her to feel some release and then refer back to her mood tracker to see the progress in her mental health, identify any trends or patterns, and reflect on any changes. This gave her encouragement that her mental health was improving when several months were compared, even if, in the moment, it did not feel this way. She also found the finished mood trackers visually appealing with the different shades of colours complementing each other, which helped encourage her to continue tracking and that was beneficial for her mood. Gabriella explained that the whole practice of bullet journaling was helpful for her mental health, not just the specific tracking of her moods. She found bullet journaling allowed her to be “super creative”, which was something she enjoyed and that helped her feel more positive. Gabriella’s bullet journal also allowed her to feel more organized, giving her space to plan a schedule and have to-do lists. She explained that physically ticking off events on her schedule and items on her to-do lists was satisfying and constructive for her mental health. The bullet journal was then a physical reminder of her productivity and creativity, which she could revisit to relive the good emotions she had experienced. 

## 4. Discussion

The experiences shared by Josie, Amelia, and Gabriella highlight the potential role for self-directed creative literacy practices in managing mental health for young people while studying. 

For both Josie and Gabriella, their engagement with literacy and creative practices provided relaxation during times of stress and anxiety. Previous literature on reading for pleasure identified relaxation as a key benefit for mental health [3,54,55,58,88]. This relaxation partially comes from reading being an enjoyable activity, as it clearly was for Josie, but also from the engagement with an interesting and diverting narrative [3]. Billington et al. suggested that in narrative fiction “the future takes care of itself” (p. 30), which allowed readers to relax and enjoy the in-world passage of time and events. In previous literature, relaxation has also been linked to the immersive nature of fiction reading [89]. Josie described feeling invested in the fictional worlds she was reading about and these worlds providing an escape from her current situation, similar to the importance placed on escapism by, for example, Nell’s participants [58]. The role of relaxation and immersion has been emphasized in previous literature on bibliotherapy and facilitated interventions [3,57,90]; Josie’s experiences suggest that self-directed reading for pleasure can also generate similar relaxation immersion. 

Josie’s experiences with reading for her mental health also highlighted how creative literacy practices interacted with practices of identity. Josie felt proud of being a bookworm and liked portraying this image of herself in public, perhaps linked to the wider societal value placed on being successful at valued literacy practices such as reading novels [85]. Her use of literacy in this way demonstrates how the ways that people engage with literacy “provide an area for constructing and performing identities” (p. 63, [91]). Engaging in creative literacy practices that promote positive self-esteem and in which students have pride may be an effective strategy for students trying to manage their mental health while at university, and is an area that warrants further research. 

For Amelia, writing poetry about her mental health experiences provided emotional catharsis and allowed her to process her emotions and gain new perspectives. This aligns with the experiences of Deveney and Lawson’s participants, who also reported feeling emotional catharsis when writing creatively about troubling emotions or experiences [1]. They described how their participants were able to reach a more “objective, detached or analytical perspective” (p. 6) by thinking about how to articulate their emotions in writing, allowing them to “create a structure out of what had hitherto been emotional chaos” (p. 7, [1]). Amelia’s experiences demonstrate that this kind of emotional processing through writing is possible for young people engaging with their own self-directed creative writing practices outside of the facilitated group setting described by Deveney and Lawson [1], or the therapeutic settings described by researchers working in the field of poetry therapy [70,71]. 

The aesthetic element of her creative writing was also helpful for Amelia, allowing her to see her difficult experiences as beautiful through the literary techniques and imagery she was using. This fits with previous work on creative writing for mental health, where the use of literary techniques has been described as a process of sense and meaning making, which allows writers to find meaning and value in distressing emotions or trauma [1,2,67,69,92]. Aesthetics also played a role in Gabriella’s use of bullet journals, with the visual expression of her moods in a creative way allowing her see her progress visually and helping her feel positive about her mental health. Both Amelia and Gabriella’s experiences show that self-directed creative literacy practices that provide students the opportunity to represent their experiences, emotions, and mental health in aesthetically pleasing textual or visual ways may be helpful for them in making sense of their difficulties and feeling positive towards their mental health. 

The possibilities for reflection through creative literacy practices was also apparent in both Amelia’s creative writing and Gabriella’s bullet journaling. Amelia revisited her poems after she had written them and refined them to better articulate her thoughts and feelings. Through this revisiting and refining, Amelia reflected on her mental health and progress she had made with her mental health, understanding her mental health better through the more objective perspective provided as part of the refining process. Amelia’s experiences echo the experiences of Deveney and Lawson’s participants, who found that refining their writing enabled them to achieve a more objective and critical perspective on their emotions, which helped them to feel more in control [1]. Gabriella used the mood tracker she drew and completed as part of her bullet journal to track her moods and reflect back over previous months to see how the pattern of her moods had changed. Previous research on bullet journals has identified self-tracking and self-reflection as key aspects of the practice [76,77]. Gabriella’s experiences highlight how both self-tracking and self-reflection through bullet journaling can provide positive effects for a student’s mental health, a relationship not fully explored in previous research. 

For all three students highlighted in this paper, the creative literacy practices they engaged in were practices they had been engaging with for some time before arriving at university and were practices they enjoyed and chose for themselves. Levine et al.’s research on reading for pleasure in university students emphasized that reading was most beneficial for mental health when it was volitional—freely chosen by the students as a practice they wanted to do [53]. The enjoyment that Josie, Amelia, and Gabriella felt from their practices was clear in the way they spoke about them. Previous research on students’ priorities for mental health support has identified that students want more information and support in using strategies for managing their mental health [28,41]. The experiences of the students discussed in this paper suggest that information and support should be particularly provided to enable students to continue engaging in creative literacy practices that they enjoy and choose. The previous literature on literacy and mental health suggests that group facilitation can be effective in introducing individuals to the practices of reading and writing for mental health [1,57,67,90]. Future research should focus on exploring such group facilitation in the context of university support services, as well as exploring how best to support self-directed practices. 

### 4.1. Research Limitations 

This paper discusses a small-scale qualitative study of the experiences of students with mental-health conditions. This approach may not be generalizable in the same way as a large quantitative study, but it was appropriate given the exploratory focus of the work. The participants were self-selected due to the use of passive recruitment and thus were those who had some existing interest in discussing mental health and their experiences of managing while at university. They were all students who were generally coping with their mental health and were not in active crisis during their participation. Their experiences may not be the same as those students who are not interested in the management of mental health or those who are actively in mental-health crisis.

### 4.2. Implications

The experiences shared by the students in this paper demonstrate how creative literacy practices can help students in managing their mental health. Mental health support providers could provide students with guidance and support in engaging in creative literacy practices. This could include running groups for students to try creative writing or bullet journaling or facilitating student book groups. For researchers, this paper demonstrates how the creative literacy practices of young people with mental-health conditions are an area rich for further study. Further research could include intervention studies or work with particular groups, such as international students or neurodivergent students.

## 5. Conclusions

These students, Josie, Amelia, and Gabriella, are case studies that demonstrate the potential role of creative literacy practices in managing mental health and promoting wellbeing while at university. Through reading, creative writing, and bullet journaling, the students found relaxation and immersion, were able to release and process emotions, and reflect on their mental health. These creative literacy practices also provided opportunities for drawing on supportive relationships, building positive self-esteem, and recording and tracking mental health progress. The volitional and self-directed nature of these practices was key for the mental health benefit, supporting previous research on students’ practices for supporting their mental health. The current situation in student mental health in the UK means that self-directed mental-health management strategies are more important than ever. Creative literacy practices represent self-directed mental-health management practices that can provide benefits for students and help address the ever-growing demand for support faced by institutions.

## Figures and Tables

**Table 1:** Interview plans for each interview stage.

Initial Guided Interview	Semi-Structured Interview 1	Semi-Structured Interview 2
Course and studies Settling in at university Non-academic interests College Support from university services Support from NHS services Previous experience of mental health before university	Process of accessing support Engagement with therapy providers Mental health self-care Hobbies and interests Social media Mood tracking Information seeking Medication Academic work	Leaflets and information texts Therapy exercises Forms and paperwork Communication with providers Medication organisation Reading for information Use of internet for mental health

**Table 2:** Participant details.

Participant Name	Gender	Age Group	Level of Study	Area of Study	Home Area	Mental-Health Condition
Laura	F	18–21	UG	Sciences	North of England	Anxiety and depression
Magda	F	21–24	PG	Social Sciences	Southern Europe	Anxiety and depression
Lucy	F	18–21	UG	Management	South of England	Eating disorder, anxiety and depression
Jordan	NB	21–24	PG	Social Sciences	East Asia	Depression
Amelia	F	18–21	UG	Humanities	North of England	Anxiety and depression
Rebecca	F	18–21	UG	Humanities	North of England	Anxiety and depression
Gabriella	F	18–21	UG	Arts	Midlands	Anxiety and depression
Sasha	F	18–21	UG	Sciences	South of England	Depression
Josie	F	18–21	UG	Social Sciences	North of England	Anxiety and depression
Charles	M	21–24	UG	Social Sciences	East Asia	Anxiety and depression
Eva	F	18–21	UG	Sciences	Central Europe	Personality disorder, eating disorder, anxiety, and depression

## Data Availability

The data presented in this study are available on request from the corresponding author. The data are not publicly available due to the small sample size and potential for identification.

## References

[1] Deveney C., Lawson P. (2021). Writing Your Way to Well-Being: An IPA Analysis of the Therapeutic Effects of Creative Writing on Mental Health and the Processing of Emotional Difficulties. Couns. Psychother. Res..

[2] Costa A.C., Abreu M.V. (2018). Expressive and Creative Writing in the Therapeutic Context: From the Different Concepts to the Development of Writing Therapy Programs. Psychologica.

[3] Billington J., Dowrick C., Hamer A., Robinson J., Williams C. (2010). An Investigation into the Therapeutic Benefits of Reading in Relation to Depression and Well-Being.

[4] McCulliss D. (2012). Bibliotherapy: Historical and Research Perspectives. J. Poet. Ther..

[5] Dingle G.A., Williams E., Jetten J., Welch J. (2017). Choir Singing and Creative Writing Enhance Emotion Regulation in Adults with Chronic Mental Health Conditions. Br. J. Clin. Psychol..

[6] Office for Students (2019). Mental Health: Are All Students Being Properly Supported? Insight.

[7] Raddi G. (2019). Universities and the NHS Must Join Forces to Boost Student Mental Health. The Guardian.

[8] (2021). BBC News Student Watchdog Concerned about Mental Health Help. BBC News.

[9] Cant S. (2018). Hysteresis, Social Congestion and Debt: Towards a Sociology of Mental Health Disorders in Undergraduates. Soc. Theory Health.

[10] Thorley C. (2017). Not by Degrees: Improving Student Mental Health in the UK’s Universities.

[11] HESA Who’s Studying in HE?|HESA. https://www.hesa.ac.uk/data-and-analysis/students/whos-in-he#numbers.

[12] McLafferty M., Lapsley C.R., Ennis E., Armour C., Murphy S., Bunting B.P., Bjourson A.J., Murray E.K., O’Neill S.M. (2017). Mental Health, Behavioural Problems and Treatment Seeking among Students Commencing University in Northern Ireland. PLoS ONE.

[13] Farrell S.M., Molodynski A., Cohen D., Grant A.J., Rees S., Wullshleger A., Lewis T., Kadhum M. (2019). Wellbeing and Burnout among Medical Students in Wales. Int. Rev. Psychiatry.

[14] Bewick B., Koutsopoulou G., Miles J., Slaa E., Barkham M. (2010). Changes in Undergraduate Students’ Psychological Well-being as They Progress through University. Stud. High. Educ..

[15] Topham P., Moller N. (2011). New Students’ Psychological Well-Being and Its Relation to First Year Academic Performance in a UK University. Couns. Psychother. Res..

[16] Macaskill A. (2012). The Mental Health of University Students in the United Kingdom. Br. J. Guid. Couns..

[17] Chen T., Lucock M. (2022). The Mental Health of University Students during the COVID-19 Pandemic: An Online Survey in the UK. PLoS ONE.

[18] Richardson T., Elliott P., Roberts R., Jansen M. (2017). A Longitudinal Study of Financial Difficulties and Mental Health in a National Sample of British Undergraduate Students. Community Ment. Health J..

[19] Barden N., Caleb R. (2019). Student Mental Health and Wellbeing in Higher Education: A Practical Guide.

[20] Cage E., Jones E., Ryan G., Hughes G., Spanner L. (2021). Student Mental Health and Transitions into, through and out of University: Student and Staff Perspectives. J. Furth. High. Educ..

[21] Johnson D.R., Soldner M., Leonard J.B., Alvarez P., Inkelas K.K., Rowan-Kenyon H.T., Longerbeam S.D. (2007). Examining Sense of Belonging among First-Year Undergraduates from Different Racial/Ethnic Groups. J. Coll. Stud. Dev..

[22] Thurber C.A., Walton E.A. (2012). Homesickness and Adjustment in University Students. J. Am. Coll. Health J. Ach..

[23] Collins H., Dailey-Strand C., Callaghan D. (2021). ‘Those First Few Months Were Horrible’: Cross-Cultural Adaptation and The J-Curve In The International Student Experience In The UK And Norway. J. Comp. Int. High. Educ..

[24] Beiter R., Nash R., McCrady M., Rhoades D., Linscomb M., Clarahan M., Sammut S. (2015). The Prevalence and Correlates of Depression, Anxiety, and Stress in a Sample of College Students. J. Affect. Disord..

[25] Cage E., Stock M., Sharpington A., Pitman E., Batchelor R. (2020). Barriers to Accessing Support for Mental Health Issues at University. Stud. High. Educ..

[26] Cramp A., Lamond C., Coleyshaw L., Beck S. (2012). Empowering or Disabling? Emotional Reactions to Assessment amongst Part-Time Adult Students. Teach. High. Educ..

[27] Arnett J.J. (2014). Emerging Adulthood: The Winding Road from the Late Teens through the Twenties.

[28] Sampson K., Priestley M., Dodd A.L., Broglia E., Wykes T., Robotham D., Tyrrell K., Vega M.O., Byrom N.C. (2022). Key Questions: Research Priorities for Student Mental Health. BJPsych Open.

[29] Stoll N., Yalipende Y., Byrom N.C., Hatch S.L., Lempp H. (2022). Mental Health and Mental Well-Being of Black Students at UK Universities: A Review and Thematic Synthesis. BMJ Open.

[30] Campbell F., Blank L., Cantrell A., Baxter S., Blackmore C., Dixon J., Goyder E. (2022). Factors That Influence Mental Health of University and College Students in the UK: A Systematic Review. BMC Public Health.

[31] Cvetkovski S., Reavley N.J., Jorm A.F. (2012). The Prevalence and Correlates of Psychological Distress in Australian Tertiary Students Compared to Their Community Peers. Aust. N. Z. J. Psychiatry.

[32] Cvetkovski S., Jorm A.F., Mackinnon A.J. (2019). An Analysis of the Mental Health Trajectories of University Students Compared to Their Community Peers Using a National Longitudinal Survey. Stud. High. Educ..

[33] Tabor E., Patalay P., Bann D. (2021). Mental Health in Higher Education Students and Non-Students: Evidence from a Nationally Representative Panel Study. Soc. Psychiatry Psychiatr. Epidemiol..

[34] Broglia E., Millings A., Barkham M. (2018). Challenges to Addressing Student Mental Health in Embedded Counselling Services: A Survey of UK Higher and Further Education Institutions. Br. J. Guid. Couns..

[35] Royal College of Psychiatrists (2011). Mental Health of Students in Higher Education.

[36] Batchelor R., Pitman E., Sharpington A., Stock M., Cage E. (2020). Student Perspectives on Mental Health Support and Services in the UK. J. Furth. High. Educ..

[37] Prince J.P. (2015). University Student Counseling and Mental Health in the United States: Trends and Challenges. Ment. Health Prev..

[38] Mair D., Mair D. (2016). The Rise and Rise of Higher Education and Therapeutic Culture. Short-Term Counselling in Higher Education.

[39] Coren A., Mair D. (2016). Short-Term Therapy. Short-Term Counselling in Higher Education.

[40] de Pury J., Dicks A. (2020). Stepchange: Mentally Healthy Universities.

[41] Priestley M., Broglia E., Hughes G., Spanner L. (2022). Student Perspectives on Improving Mental Health Support Services at University. Couns. Psychother. Res..

[42] Barton D., Hamilton M., Barton D., Hamilton M., Ivanič R. (2000). Literacy Practices. Situated Literacies.

[43] Papen U., Walters S. (2008). Literacy, Learning and Health. Research Report.

[44] Papen U. (2009). Literacy, Learning and Health—A Social Practices View of Health Literacy. Lit. Numeracy Stud..

[45] Muro A. (2006). What Is Health Literacy.

[46] Peach E. (2022). Mental Health Literacy Practices: An Exploration of How Students with Mental Health Conditions Use Literacy to Navigate Support Systems, Manage Their Mental Health, and Engage with Being a Student. Ph.D. Thesis.

[47] Jack S.J., Ronan K.R. (2008). Bibliotherapy: Practice and Research. Sch. Psychol. Int..

[48] Lucas C.V., Soares L. (2013). Bibliotherapy: A Tool to Promote Children’s Psychological Well-Being. J. Poet. Ther..

[49] Collins V.J., Dargan I.W., Walsh R.L., Merga M.K. (2022). Teachers’ Perceptions of the Benefits and Challenges of a Whole-School Reading for Pleasure Program. Issues Educ. Res..

[50] Burnett C., Merchant G. (2018). Affective Encounters: Enchantment and the Possibility of Reading for Pleasure. Literacy.

[51] Gagen-Spriggs K. (2020). An Investigation into the Reasons Students Read for Pleasure. Sch. Libr. Worldw..

[52] Howard V. (2011). The Importance of Pleasure Reading in the Lives of Young Teens: Self-Identification, Self-Construction and Self-Awareness. J. Librariansh. Inf. Sci..

[53] Levine S.L., Cherrier S., Holding A.C., Koestner R. (2022). For the Love of Reading: Recreational Reading Reduces Psychological Distress in College Students and Autonomous Motivation Is the Key. J. Am. Coll. Health.

[54] Merga M. (2017). Becoming a Reader: Significant Social Influences on Avid Book Readers. Sch. Libr. Res..

[55] Clark C., Picton I. (2020). Children and Young People’s Reading in 2020 before and during the COVID-19 Lockdown.

[56] Usherwood B., Toyne J. (2002). The Value and Impact of Reading Imaginative Literature. J. Librariansh. Inf. Sci..

[57] Dowrick C., Billington J., Robinson J., Hamer A., Williams C. (2012). Get into Reading as an Intervention for Common Mental Health Problems: Exploring Catalysts for Change. Med. Humanit..

[58] Nell V. (1988). The Psychology of Reading for Pleasure: Needs and Gratifications. Read. Res. Q..

[59] Prater M.A., Johnstun M.L., Dyches T.T., Johnstun M.R. (2006). Using Children’s Books as Bibliotherapy for At-Risk Students: A Guide for Teachers. Prev. Sch. Fail. Altern. Educ. Child. Youth.

[60] Pennebaker J.W., Beall S.K. (1986). Confronting a Traumatic Event: Toward an Understanding of Inhibition and Disease. J. Abnorm. Psychol..

[61] Hunt C. (2000). Therapeutic Dimensions of Autobiography in Creative Writing.

[62] Schwartz L., Drotar D. (2004). Effects of Written Emotional Disclosure on Caregivers of Children and Adolescents with Chronic Illness. J. Pediatr. Psychol..

[63] Cooper P. (2013). Writing for Depression in Health Care. Br. J. Occup. Ther..

[64] Pennebaker J.W. (2000). The Effects of Traumatic Disclosure on Physical and Mental Health: The Values of Writing and Talking about Upsetting Events. Posttraumatic Stress Intervention: Challenges, Issues, and Perspectives.

[65] Pennebaker J.W., Evans J.F. (2014). Expressive Writing: Words That Heal.

[66] Jess-Cooke C. (2015). Should Creative Writing Courses Teach Ways of Building Resilience?. New Writ..

[67] King R., Neilsen P., White E. (2013). Creative Writing in Recovery from Severe Mental Illness. Int. J. Ment. Health Nurs..

[68] Kempler N.Z. (2003). Finding Our Voice through Poetry and Psychotherapy. J. Poet. Ther..

[69] Croom A.M. (2015). The Practice of Poetry and the Psychology of Well-Being. J. Poet. Ther..

[70] Mazza N. (1999). Poetry Therapy: Interface of the Arts and Psychology.

[71] Anderson R.N. (1999). The Healing Powers of Creativity: Using Poetry in Psychotherapy. Individ. Psychol..

[72] Tamura H. (2001). Poetry Therapy for Schizophrenia: A Linguistic Psychotherapeutic Model of Renku (Linked Poetry). Arts Psychother.-Art Psychother..

[73] Houlding S., Holland P. (1988). Contributions of a Poetry Writing Group to the Treatment of Severely Disturbed Psychiatric Inpatients. Clin. Soc. Work J..

[74] Dubrasky D., Sorensen S., Donovan A., Corser G. (2019). “Discovering Inner Strengths”: A Co-Facilitative Poetry Therapy Curriculum for Groups. J. Poet. Ther..

[75] Sharma D. (2021). Reading and Rewriting Poetry on Life to Survive the COVID-19 Pandemic. J. Poet. Ther..

[76] Tholander J., Normark M. Crafting Personal Information-Resistance, Imperfection, and Self-Creation in Bullet Journaling. Proceedings of the 2020 CHI Conference on Human Factors in Computing Systems.

[77] Ayobi A., Sonne T., Marshall P., Cox A.L. (2018). Flexible and Mindful Self-Tracking: Design Implications from Paper Bullet Journals. Proceedings of the 2018 CHI Conference on Human Factors in Computing Systems.

[78] Peach E. (2021). Using Skype to Research Literacy Practices: Providing Opportunities for Participants with Mental Health Conditions to Share Their Experiences. Literacy.

[79] Gillen J., Ho W.S., Tusting K. (2019). Literacy Studies. The Routledge Handbook of Linguistic Ethnography.

[80] Olson K. (2016). Essentials of Qualitative Interviewing.

[81] Rubin H., Rubin I. (2005). Qualitative Interviewing: The Art of Hearing Data.

[82] Evaer Record Skype Call with High Quality Video Recorder|Evaer. https://www.evaer.com/.

[83] Braun V., Clarke V. (2006). Using Thematic Analysis in Psychology. Qual. Res. Psychol..

[84] QSR International Pty Ltd. NVivo 11 Plus 2015. https://www.bloomberg.com/profile/company/1309654D:AU.

[85] McCarthey S.J. (2001). Identity Construction in Elementary Readers and Writers. Read. Res. Q..

[86] McCarthey S.J., Moje E.B. (2002). Identity Matters. Read. Res. Q..

[87] Tatum B.D. (2017). Why Are All the Black Kids Sitting Together in the Cafeteria? Revised..

[88] Dungworth N., Grimshaw S., Mcknight C., Morris A. (2004). Reading for Pleasure?: A Summary of the Findings from a Survey of the Reading Habits of Year 5 Pupils. New Rev. Child. Lit. Librariansh..

[89] Cohen L.J. (1993). The Therapeutic Use of Reading: A Qualitative Study. J. Poet. Ther..

[90] Billington J., Davis P., Farrington G. (2013). Reading as Participatory Art: An Alternative Mental Health Therapy. J. Arts Communities.

[91] Merchant G., Carrington V. (2009). Literacy and Identity. Literacy.

[92] Lepore S.J., Smyth J.M. (2002). The Writing Cure: How Expressive Writing Promotes Health and Emotional Well-Being.

